# Usefulness of white blood cell count to mean platelet volume ratio in the prediction of SYNTAX score in patients with non-ST elevation myocardial infarction

**DOI:** 10.12669/pjms.35.3.1017

**Published:** 2019

**Authors:** Serkan Sivri, Erdogan Sokmen, Mustafa Celik, Sinan Cemgil Ozbek, Alp Yildirim, Yalcin Boduroglu

**Affiliations:** 1 *Dr. Serkan Sivri, Department of Medicine, Ahi Evran University Training and Research Hospital, Kirsehir, Turkey*; 2 *Dr. Erdogan Sokmen, Department of Medicine, Ahi Evran University Training and Research Hospital, Kirsehir, Turkey*; 3 *Dr. Mustafa Celik, Department of Medicine, Ahi Evran University Training and Research Hospital, Kirsehir, Turkey*; 4 *Dr. Sinan Cemgil Ozbek, Department of Medicine, Ahi Evran University Training and Research Hospital, Kirsehir, Turkey*; 5 *Dr. Alp Yildirim, Department of Medicine, Ahi Evran University Training and Research Hospital, Kirsehir, Turkey*; 6 *Dr. Yalcin Boduroglu, Department of Medicine, Ahi Evran University Training and Research Hospital, Kirsehir, Turkey*

**Keywords:** Acute coronary syndrome, Coronary atherosclerosis, SYNTAX score, White blood cell count to mean platelet volume ratio

## Abstract

**Objective:**

White blood cell (WBC) count to mean platelet volume (MPV) ratio (WMR) is associated with major adverse cardiovascular events in patients with non-ST elevation acute coronary syndrome (NSTEMI). We aimed to compare WMR between NSTEMI patients and matched-controls and to evaluate its predictive value on SYNTAX score.

**Methods:**

Total 175 patients with NSTEMI and 160 age and co-morbidity matched subjects were recruited in our study. WMR was compared between the patient and control groups. The patient group was further subdivided into 3 tertiles according to SYNTAX scores as follows: low SYNTAX score tertile (score ≤22, 141 patients); intermediate SYNTAX score tertile (score between 23 and 32, 20 patients); and, high SYNTAX score tertile (score ≥33, 14 patients). WMR was further assessed among the tertiles.

**Results:**

WMR was significantly greater in the patient group compared to the control group (p<0,001). WMR among low, intermediate and high score tertiles were calculated to be 890±26, 1090±042 and 1500±65, respectively (p <0,001). In receiver operating characteristics (ROC) analysis, WMR >960 predicted a SYNTAX score ≥23 with 80.6% sensitivity and 67.6% specificity (AUC: 0.756; 95% CI: 0.685 - 0.818; p <0.0001) and a WMR >1360 predicted a SYNTAX score ≥33 with 71.4% sensitivity and 93% specificity (AUC: 0.840; 95%CI: 0.777 - 0.892; p <0.0001).

**Conclusions:**

WMR value was significantly elevated in NSTEMI patients, compared to controls. Higher WMR was associated with greater SYNTAX score in patients with NSTEMI. WMR may be used to predict severity of the CAD and to implement risk stratification in patients with NSTEMI.

## INTRODUCTION

Acute coronary syndrome (ACS) is a disease characterized in most of the cases by rupture of atherosclerotic plaque and subsequent complete or incomplete thrombosis of the coronary arteries.^1^ It has still persisted to be the most common reason for death worldwide, despite ongoing amelioration in the rate of mortality.^2^

Previous studies have reported increased admission white blood cell (WBC) count to be a robust predictor of morbidity and mortality in patients with ACS.^3^,^4^ Mean platelet volume (MPV) has long been a byword for the marker of platelet activation. Increased MPV readily translates into more active and thrombogenic platelets than smaller ones.^5^,^6^ Accordingly, both increased MPV and WBC count on admission was documented previously to have a statistical significance in the prediction of impaired reperfusion and mortality in patients with ACS.^7^,^8^

In the present study, we intended to assess WMR, has emerged as a novel inflammatory marker, on admission in patients with NSTEMI, and its further association with SYNTAX score.

## METHODS

A total of 175 consecutive patients who were admitted to the emergency department of our hospital between July 2017 - January 2018 and diagnosed with NSTEMI were recruited in our study. The exclusion criteria were set as follows: lack of patient consent, previously known CAD, significant valvular heart disease or valve surgery, chronic renal failure, recent surgery, use of steroid or non-steroid anti-inflammatory treatment. The diagnosis of NSTEMI was confirmed if patients had typical angina pain lasting 30 minutes, elevated cardiac troponin levels and no ST segment elevation detectable in the electrocardiogram (ECG). Moreover, a total of 160 subjects with no previous history of CAD who were matched regarding age and co-morbidities were employed as control group. Blood sample was obtained on admission from each patient for the measurement of complete blood count, troponin levels, liver and kidney function tests and bleeding profile. Twelve hour fasting serum lipid profiles were measured by standard enzymatic methods. The study was approved local ethics committee and informed consents were obtained from all the participants.

Coronary Angiography and Interventions:] All patients were treated by complying with the recommendations of NSTEMI guideline.^9^ Coronary angiography was performed in all patients using the standard techniques. Glycoprotein IIb/IIIa inhibitor (Tirofiban) was administered to the patients in the catheterization laboratory at the operator’s discretion. Decision regarding the implementation of percutaneous coronary intervention (PCI), coronary artery bypass grafting (CABG) surgery or medical treatment was given by a heart team comprising two cardiologists and one cardiovascular surgeon. All PCIs performed in eligible patients were performed using the standard clinical practice and choice between the alternatives of drug-eluting stent or bare metal stent was at the operator’s discretion. In most of the patients, stenting of infarct-related artery was successfully fulfilled. Medical treatment or CABG surgery was applied to the rest of the patients.

Assessment of cineangiographic view was performed using Axiom (Siemens Medical Solution, Erlangen, Germany) workstation by two experienced cardiologist blinded to the study data. Each lesion with a diameter stenosis ≥50% in coronary vessels ≥1.5 mm in diameter was scored using the online SYNTAX score calculator.^10^ If the cardiologists disagree about the lesions, the ultimate score was decided by averaging the scores calculated by each cardiologist.

### Statistical analysis

SPSS v.22.0 software for Windows (SPSS Inc. Chicago, llinois, USA) was used for the statistical analysis. Continuous variables were expressed as mean± standard deviation (SD), while categorical variables were expressed in numbers and percentages. The normal distribution of the data was evaluated by Kolmogorov-Smirnov and Shapiro-Wilk test. Nonparametric variables between groups were compared with the Mann-Whitney U test or Student’s test. The patient group population was further divided into tertiles on the basis of SYNTAX scores calculated. One-way analysis of variance (ANOVA) test was used for continuous variables, whereas categorical variables were compared with the chi-square test among SYNTAX score tertiles. Receiver operating characteristic (ROC) curve analysis was used to detect the sensitivity and specificity of WMR and its cutoff values in the prediction of SYNTAX score. P value <0.05 was accepted to be statistically significant.

## RESULTS

The patient group consisted of 175 NSTEMI patients (mean age: 60.1±12.0 years). The control group consisted of 160 subjects (mean age: 59.4±9.6 years). Demographic, echocardiographic and biochemical characteristics of all study population was presented in Table-I. There was no statistically significant difference with regard to age (p=0.571) and such other co-morbid factors as diabetes mellitus (p=0.055), hypertension (p=0.140), chronic obstructive pulmonary disease (p=0,186) and obesity (p=0.261) between two groups. Total cholesterol and low density lipoprotein levels were similar between the groups (p=0.158 and p=0.105, respectively), in contrast, high density lipoprotein level was significantly lower in patient group (41.10±14.67 mg/dL vs. 45.53±13.09 mg/dL, p=0.001). WBC and neutrophil counts were higher in the patients group compared to the control group (9.14±3.54 x10^3^/uL vs. 8.03±2.14 x10^3^/uL, p=0.002 and 6.05±2.86 vs. 4.74±1.69, p<0,001; respectively). Lymphocyte and platelet counts were significantly lower in patient group (2.23±1.24 x10^3^/uL vs. 2.50±0.69 x10^3^/uL, p<0.001 and 220.22±63.35 x10^3^/uL vs. 266.87±62.72 x10^3^/uL, p<0.001; respectively), while creatinine and blood glucose levels were significantly lower in control group (0.81±0.83 mg/dL vs. 1.07±0.77 mg/dL, p<0.001 and 104.37±56.51 mg/dL vs. 155.28±78.26 mg/dL, p<0.001; respectively). MPV was lower in patient group compared to the control group (9.68±1.34 fL and 10.71±0.94 fL, respectively; p<0.001). In addition, WMR was found to be significantly greater in the patient group compared to the control group (960±37 and 760±21, respectively; p<0.001). As for the echocardiographic parameters, they were similar between two groups except left ventricular ejection fraction which was significantly lower in the patient group compared to the control group (51.77±12.25% and 62.95±5.75%, respectively; p<0.001). Median SYNTAX score of the patient group was 11.79 (0-38.5). Moreover, the patient group was further subdivided into 3 tertiles according to the SYNTAX scores as follows: low SYNTAX score tertile (score ≤22, 141 patients); intermediate SYNTAX score tertile (score between 23 and 32, 20 patients); and, high SYNTAX score tertile (score ≥33, 14 patients) (Table-II). WMR among low, intermediate and high score tertiles were calculated to be 890±26, 1090±042 and 1500±65, respectively (p <0.001). As can be seen from the Table-II, the majority of the patient group had fitted into the low SYNTAX score tertile.

**Table-I:** Demographic, biochemical and echocardiographic characteristics of NSTEMI patient and control groups.

Variables	Patient Group (n=175)	Control Group (n=160)	P-value
Age, years	60.1±12.0	59.4±9.6	0.571
DM, n (%)	57 (32.6%)	37 (23.1%)	0.055
HT, n (%)	94 (53.7%)	73 (45.6%)	0.140
COPD, n (%)	31 (17.7%)	20 (12.5%)	0.186
Obesity, n (%)	22 (12.6%)	14 (8.7%)	0.261
SYNTAX score	11,79 (0-38.5)	-	-
WBC (x10^3^/uL)	9.14±3.54	8.03±2.14	0.002
MPV (fL)	9.68±1.34	10.71±0.94	<0.001
WMR	960±37	760±21	<0.001
Neutrophile (x10^3^/uL)	6.05±2.86	4.74±1.69	<0.001
Lymphocyte(x10^3^/uL)	2.23±1.24	2.50±0.69	<0.001
Platelet (x10^3^/uL)	220.22±63.35	266.87±62.72	<0.001
Glucose (mg/dL)	155.28±78.26	104.37±56.51	<0.001
Creatinine (mg/dL)	1.07±0.77	0.81±0.83	<0.001
Total cholesterol (mg/dL)	187.57±42.86	193.79±40.00	0.158
LDL (mg/dL)	110.20±36.44	116.61±34.69	0.105
HDL (mg/dL)	41.10±14.67	45.53±13.09	0.001
Troponin (pg/mL)	441.11 (3-10950)	-	-
CK-MB (ng/mL)	15.82 (0.40-300)	-	-
LVEDD (cm)	4.71±0.46	4.61±0.31	0.442
LVESD (cm)	3.00±0.70	2.75±0.40	0.057
EF. %	51.77±12.25	62.95±5.75	<0.001
LA (cm)	3.72±0.45	3.64±0.54	0.170

CK-MB: Creatin kinase myocardial band, COPDB: Chronic obstructive pulmonary disease,DM: diabetes mellitus, EF: Ejection fraction, HDL: High-density lipoprotein, HT: Hypertension,LDL: Low-density lipoprotein, LA: Left atrium, LVEDD: Left ventricle end-diastolic diameter, LVESD: Left ventricle end-systolic diameter, MPV: Mean platelet volume, WBC: White blood cell, WMR: White blood cell count to mean platelet volume ratio.

**Table-II:** ANOVA analysis of WMR parameter in SYNTAX score tertiles.

Variable	SYNTAX≤22 (141 patients)	SYNTAX 23-32 (20 patients)	SYNTAX≥33 (14 patients)	P-value
WMR	890±26	1090±042	1500±65	<0.001

**Fig.1:**
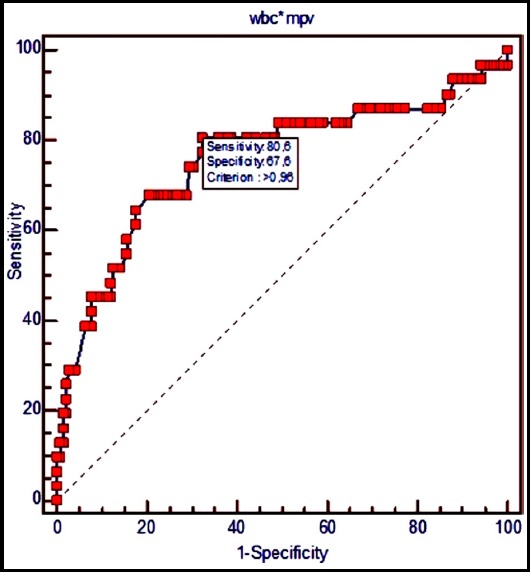
Sensitivity and specificity of WMR variable for predicting SYNTAX score ≥23.

**Fig.2:**
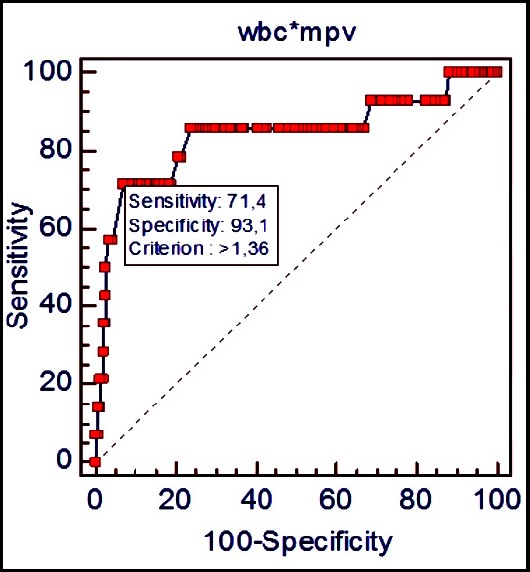
Sensitivity and specificity of WMR variable for predicting SYNTAX score ≥33.

According to ROC curve analysis, a WMR >960 showed a sensitivity of 80.6% and a specificity of 67.6% in predicting a SYNTAX score ≥23 (AUC: 0.756; 95% CI: 0.685 - 0.818; p<0.0001) (Fig.1). Furthermore, a WMR > 1360 predicted a SYNTAX score ≥33 with a sensitivity of 71.4% and a specificity of 93% (AUC: 0.840; 95% CI: 0.777 - 0.892; p <0.0001) (Fig.2).

## DISCUSSION

This study is perhaps the first to show elevated WMR in NSTEMI patients compared with age and comorbidity-matched control subjects. Moreover, our study also demonstrated that baseline WMR increased significantly as the SYNTAX score increased in patients with NSTEMI, yielding a WMR cut-off value of 960 to predict SYNTAX score ≥23 and another cut-off value of 1360 to predict SYNTAX score ≥33.

Inflammation has long been known to contribute intricately to development, progression and destabilization of atherosclerotic plaques. Platelets, leukocytes and vascular endothelial cells interact with each other through a constellation of mediators or inflammation markers operating concurrently, thereby resulting in atherosclerotic plaque rupture in the setting of ACS.^11^,^12^ In addition, WBC count and some other hematological indices such as neutrophil count, lymphocyte count, NLR, MPV, RDW and MPV to lymphocyte ratio have previously been reported to have predictive and prognostic value in the setting of ACS.^13^-^19^

MPV is among the parameters pertaining to platelet activation. Larger platelets are more active and thrombogenic. Although there are studies reporting increase in MPV in ACS patients or patients possessing cardiovascular risk factors, some others yielded conflicting results.^13^,^17^,^18^,^20^-^24^ On the other hand, MPV and platelet count were demonstrated in another study to decrease initially 3 hours after the hospital admission, followed by increase on the 3^rd^ and 7^th^ days in patients with STEMI and NSTEMI.^25^ The fact that platelet count and MPV were significantly lower in patient group than in control group may in part be explained consumption and sequestration of platelets, especially those with later volume, during the acute phase of ACS.^25^

Likewise, WMR has emerged as a novel inflammatory marker with documented efficacy in the prediction of prognosis in ACS. Dehghani MR et al.^13^ reported that WMR was stronger in predicting long-term adverse outcomes than other complete blood cell indices in patients with NSTEMI. Similarly, Cicek G et al.^19^ documented higher admission WMR to be better predicted long-term mortality compared to such complete blood count indices as MPV, RDW, PLR and NLR in patients with STEMI. In a recent study by Adam AM et al.^18^, WMR on admission was more significant positive correlation with increased short-term mortality compared to other complete blood cell indices. Additionally, their study also reported that ACS patients with WMR>1000 on admission were associated with higher rates of multi-vessel disease, higher incidence of mortality and greater Thrombolysis in Myocardial Infarction (TIMI) Score, compared to those with admission WMR<1000. Accordingly, cut-off values of 960 and 1360 to predict SYNTAX scores ≥23 and ≥33, respectively, seem quite similar to that study. SYNTAX score has been a widely accepted scoring system which is utilized to assess the disease complexity of entire coronary arterial system and helps clinicians stratify the potential patients most likely to benefit either from PCI or CABG surgery.^10^,^26^ Moreover, the higher the SYNTAX score, the worse the adverse cardiovascular outcomes and the more complicated the CAD in a particular case.^26^ Considering our study findings in this regard, it is prudent to anticipate increase in WMR values with increasing SYNTAX score and hence short and long term mortality.

### Limitations of the study

First, our study was a single center study with relatively small number of recruitment. We did not interrogate any probable relationship between other complete blood count parameters and SYNTAX scores of the patient group. We also did not compare the groups and SYNTAX score tertiles on the gender basis.

## CONCLUSION

WMR value was significantly elevated in NSTEMI patients, compared to the age and comorbidity-matched controls. Moreover, higher WMR was positively correlated with greater SYNTAX score in the setting of NSTEMI. WMR is likely to be utilized in predicting the severity of the CAD and to implement risk stratification in patients with NSTEMI. However, further studies are needed to validate our study findings.

### Author`s Contribution

**SS** conceived and designed.

**SS and ES** did statistical analysis & editing of manuscript.

**MC, SCO and AY** did data collection and manuscript writing.

**YB** did review and final approval of manuscript.
